# Supplementary material for: Double charge flips of polyamide membrane by ionic liquid-decoupled bulk and interfacial diffusion for on-demand nanofiltration
Source: Nat Commun. 2024 Mar 13;15:2282. doi: 10.1038/s41467-024-46580-6 (PMC10937904; doi:10.1038/s41467-024-46580-6)
Supplement: Supplementary file 1 — Supplementary Information [file 41467_2024_46580_MOESM1_ESM.pdf]

Supplementary Information

Double charge flips of polyamide membrane by ionic liquid-decoupled bulk and interfacial diffusion for on-demand nanofiltration

Bian-Bian Guo[1†], Chang Liu[1,2†], Cheng-Ye Zhu[1,2], Jia-Hui Xin[1], Chao Zhang[1,2,*], Hao-Cheng Yang[1,2,*], Zhi-Kang Xu[1,2,*]

[1] Key Lab of Adsorption and Separation Materials & Technologies of Zhejiang Province, MOE Engineering Research Center of Membrane and Water Treatment, Department of Polymer Science and Engineering, Zhejiang University, Hangzhou 310058, China

[2] The "Belt and Road" Sino-Portugal Joint Lab on Advanced Materials, International Research Center for X Polymers, Zhejiang University, Hangzhou 310058, China

*Corresponding authors: zhangchao7@zju.edu.cn, yanghch@zju.edu.cn, xuzk@zju.edu.cn

† The two authors contributed equally to this work.

This file includes:

Supplementary Methods

Supplementary Figures 1 to 26

Supplementary Tables 1-10

Supplementary References 61

1. Supplementary Methods

1.1. Chemicals and Materials. Polyether sulfone (PES) microfiltration substrates (pore size 0.22 μm, porosity 73.5%) were supplied by the Haiyan Delv Science and Technology Company. Ionic liquids, 1-butyl-3-methylimidazoilum tetrafluoroborate ([Bmim][BF_4]) and 1-ethyl-3-methylimidazolium tetrafluoroborate ([Emim][BF_4]) was purchased from Shanghai Dibo Chemicals Technology Co., Ltd. Trimesoyl chloride (TMC, purity >99%) was afforded by Qingdao Benzo Advanced Materials Co., Ltd. Piperazine (PIP, anhydrous), anhydrous $MgCl_2$ and LiCl were bought from Shanghai Aladdin Biochemical Technology Co., Ltd. Vitamin B12 (VB12) was provided by Sigma-Aldrich brand. Other chemicals such as inorganic salts (NaCl (99%), Na_2SO_4 (99%), $MgSO_4$ (99%)), anhydrous organic solvents (ethanol, N, N-dimethylformamide, hexane, glycerol), polyethylene glycol (PEG, M_w 200, 400, 600, 800, 1000), and sodium dodecyl sulfate (SDS) were acquired from Sinopharm Chemical Reagent Co., Ltd. All chemicals were used as received without further purification. Deionized water (18.2 MΩ·cm) was generated with the ELGA Labwater purification system (VWS Ltd., France).

12. Characterizations. The surface chemistry of polyamide membranes was characterized with Fourier transform infrared spectroscopy (FT-IR, Bruker, Nicolet 6700, USA) and X-ray photoelectron spectrometer (XPS, Thermo Scientific, K-Alpha, USA). The PA-IL nanofilm was obtained by dissolving the PES substrate in DMF and it was transferred onto a silicon wafer for XPS characterization. The water wettability of PA-IL membranes and the surface tension of the IL-water mixtures were measured with a contact angle meter (Ningbo NBSi, OSA 200, China). The viscosity of IL-water mixtures was quantified by a rotary rheometer (Thermo Scientific, HAKKE Mars 60, Germany). The surface charge property was analyzed by an electrokinetic analyzer (Anton Paar, Surpass, Austria). Surface and cross-sectional morphologies of PA-IL membranes were observed with Field emission scanning electron microscopy (FESEM, Hitachi, SU4080, Japan) and white light interferometer (Vecco, NT9100, USA). Step profiler (Bruker, DEKTAK-XT, Germany) could measure the thickness of a substrate-free polyamide nanofilm.

1.3 In-situ UV monitoring of monomer diffusion. The interfacial monomer diffusion dynamics was investigated by in-situ UV spectroscopy (Shimadzu, UV2450, Japan). A pre-installed slit on the sample stage filtrates the signal beyond the interface region. 20 g L^{-1} PIP solution of 700 μL was carefully injected into the cuvette as the polar phase, and 1000 μL pure hexane was sequentially added. A stable interface was formed. At this time, UV spectroscopy could record the PIP amount in the hexane phase in real time for 1200 s. Diffusion dynamics of TMC was also measured at the interface between 0.25 g L^{-1} TMC hexane solution and PIP-free IL/water solution. UV absorbance at the interface between hexane and PIP-free IL/water solution was as the background signal to eliminate the influence of IL.

1.4 DFT calculation. The Schotten-Baumann reaction between PIP and TMC adheres to the S_N2 mechanism, which is an elementary step. Initially, PIP and TMC were constructed with Gaussview and optimized via Gaussian 16 software package[1] at the B3LYP-D3(BJ)[2,3] level of theory with a def2-TZVP basis set[4]. Transition state structures were hypothesized based on chemical intuition and optimized utilizing the opt=TS keyword, with the correctness of the transition state structures validated through intrinsic reaction coordinate (IRC) searches to identify reactants and products (Supplementary Fig. 12). Single-point energies for the reactant complexes and transition states were calculated separately at the aforementioned computational level to obtain the reaction barrier. The TSTcalculator, developed by Dr. Tian Lu was utilized to compute reaction rate constants via transition state theory[5], while transmission coefficients were approximated using the Skodje-Truhlar method and the reaction path degeneracy was determined from rotational symmetry numbers derived from frequency calculations[6].

2. Supplementary Figures

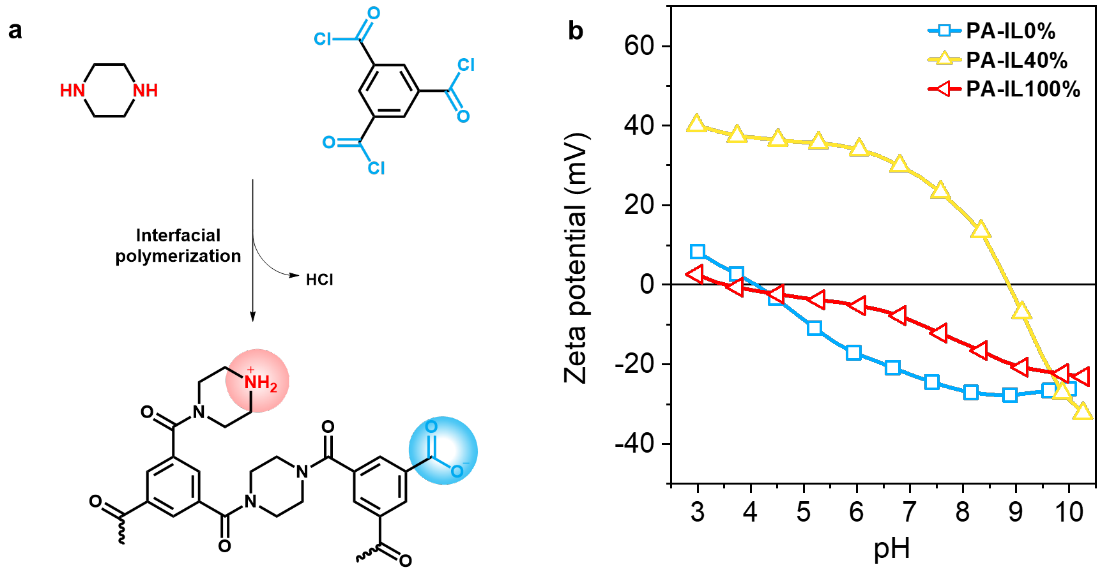

Supplementary Fig. 1 (a) Polyamide formed through the interfacial polymerization between piperazine (PIP) in ionic liquid (IL)/water mixture and trimesoyl chloride (TMC) in hexane, named PA-ILx% membrane, the suffix x% refers to the IL volume fraction in the IL/water mixture. **(b)** Zeta potential curves versus pH for pristine PA-IL0% and different PA-IL membranes.

Pristine PA-IL0% membrane is negatively charged at neutral pH as analyzed by zeta potential. However, varied IL/water mixtures enable the double charge flips of polyamide membranes, for example, the surface charge flips to be positive for PA-IL40% membrane and then displays the second flip to be negative for PA-IL100% membrane.

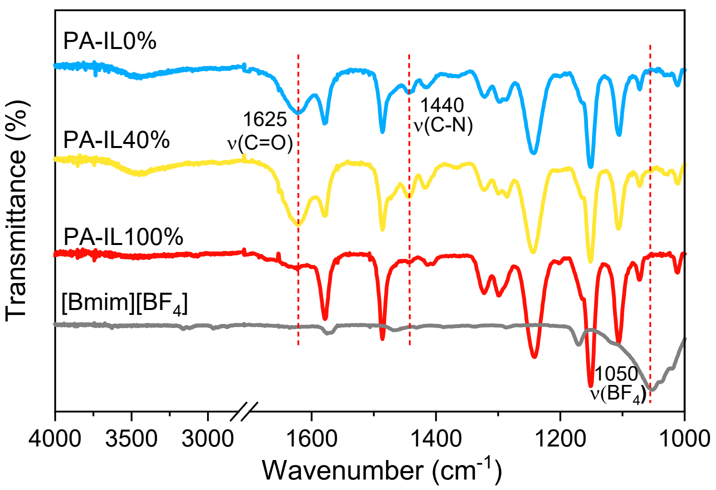

Supplementary Fig. 2 FTIR spectra of [Bmim][BF_4] and PA-IL membranes.

As shown in supplementary Fig. 2, strong amide absorption peaks in 1625 cm^{-1} (amide I, stretching C=O bond) and 1440 cm^{-1} (amide II, stretching of C-N bond) are observed, indicating the formation of polyamide nanofilms at different IL contents. Meanwhile, neglectable absorption in 1050 cm^{-1} (vibration of BF_4^-) demonstrates little residual IL in different PA-IL membranes, even at a high IL content.

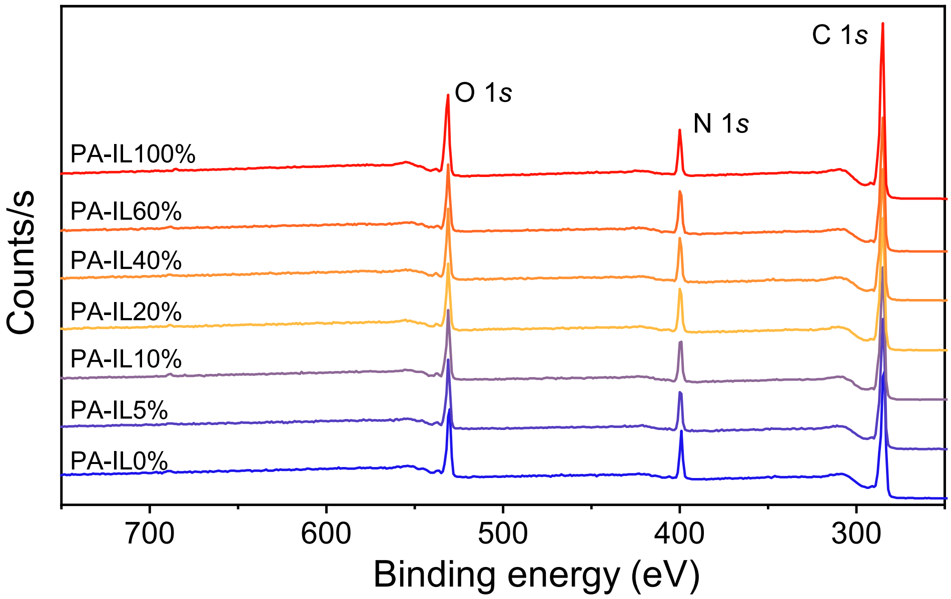

Supplementary Fig. 3 XPS spectra of a series of PA-IL membranes. These polyamide layers mainly consist of C, N and O elements. The F element from additional [Bmim][BF_4] co-solvent is as weak as baseline noise below 0.5%. All samples are DMF washed before XPS characterization.

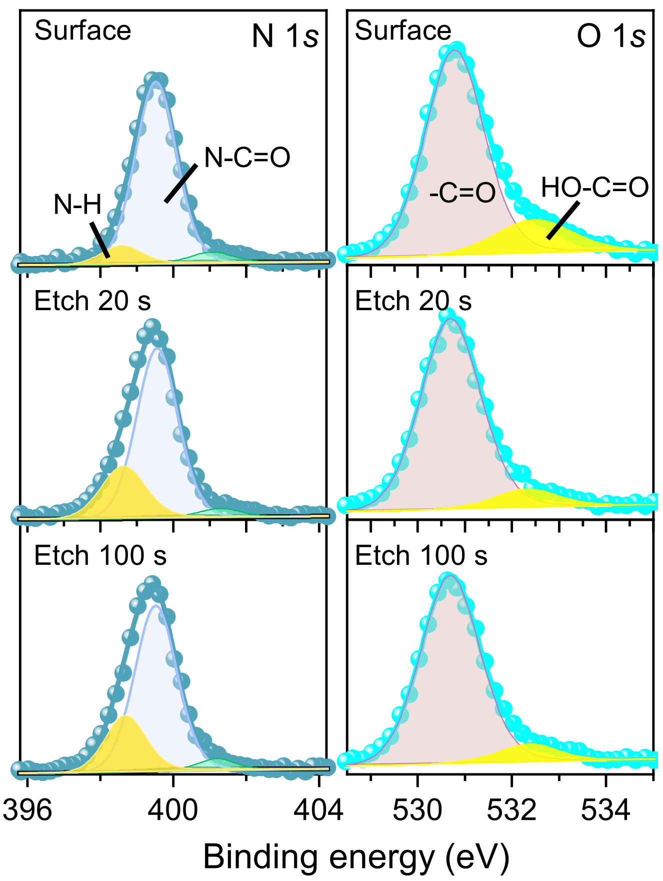

Supplementary Fig. 4 Deconvolution of N1*s* and O1*s* spectra along the polyamide profile of the PA-IL40% membrane.

Control PA-IL40% membrane was prepared as depicted in the experimental section. 8 g L^{-1} PIP dissolved in 40 v/v% IL solution was filtrated within the PES substrate under vacuum assistance and then 2.4 g L^{-1} TMC solution was added to trigger interfacial polymerization for 60 s. Following that, various post-treatments were performed on the immature PA-IL40% membrane: (1) immature polyamide membrane was immersed into DI water for complete hydrolysis of unreacted acyl chloride. (2) PA-IL40% was immersed in ethyl acetate containing 10 g L^{-1} benzoyl chloride (PhCOCl) for 24 h at room temperature for sufficient reaction with amino groups on the polyamide surface. (3) The same procedure as part (2) was conducted on the PA-IL40% membrane while the reagent PhCOCl was replaced by TMC. These three kinds of post-treated PA-IL40% membranes are different in the surface functional groups, which is revealed by the surface charge property (Supplementary Fig. 5). The water immersion treated PA-IL40% membrane is more positive because of less remained acyl chloride. On the contrary, PhCOCl-modified membrane is near-neutral and the TMC-modified membrane is negative owing to the effective grafting of acyl chloride into the polyamide network. Therefore, it is further verified the positive nature of PA-IL40% membrane comes from the inherently abundant amino groups.

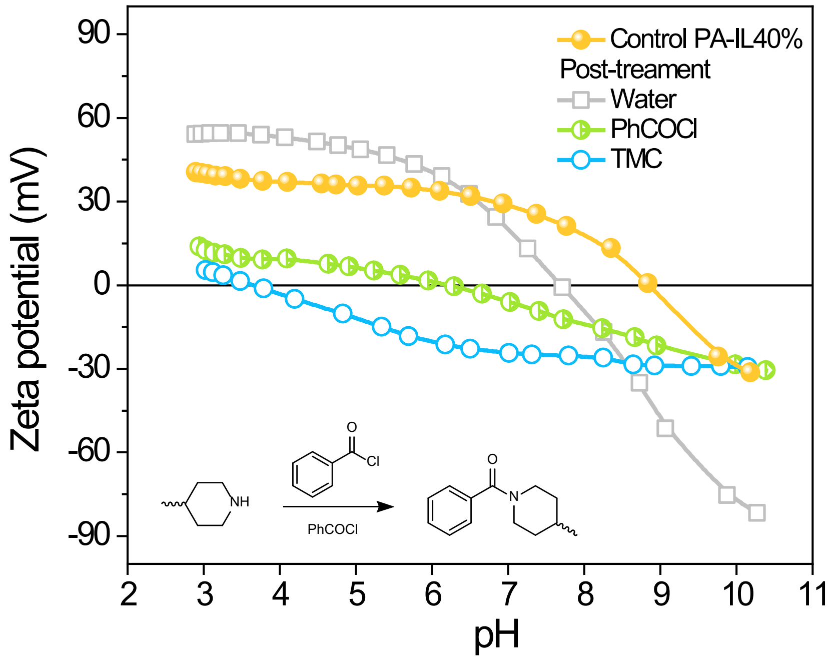

Supplementary Fig. 5 Zeta potential curves of PA-IL40% membranes post-treated with different chemicals. Insert: chemical formula of the condensation reaction between amine and benzoyl chloride (PhCOCl).

The monomer diffusion kinetics was experimentally investigated by in-situ UV-vis spectroscopy. The UV absorbance is linear with the concentration of [Bmim][BF_4], PIP, and TMC at 230 nm, 230 nm, and 290 nm, respectively. Therefore, the change of UV absorbance can be converted the diffusion amount of PIP and TMC directly.

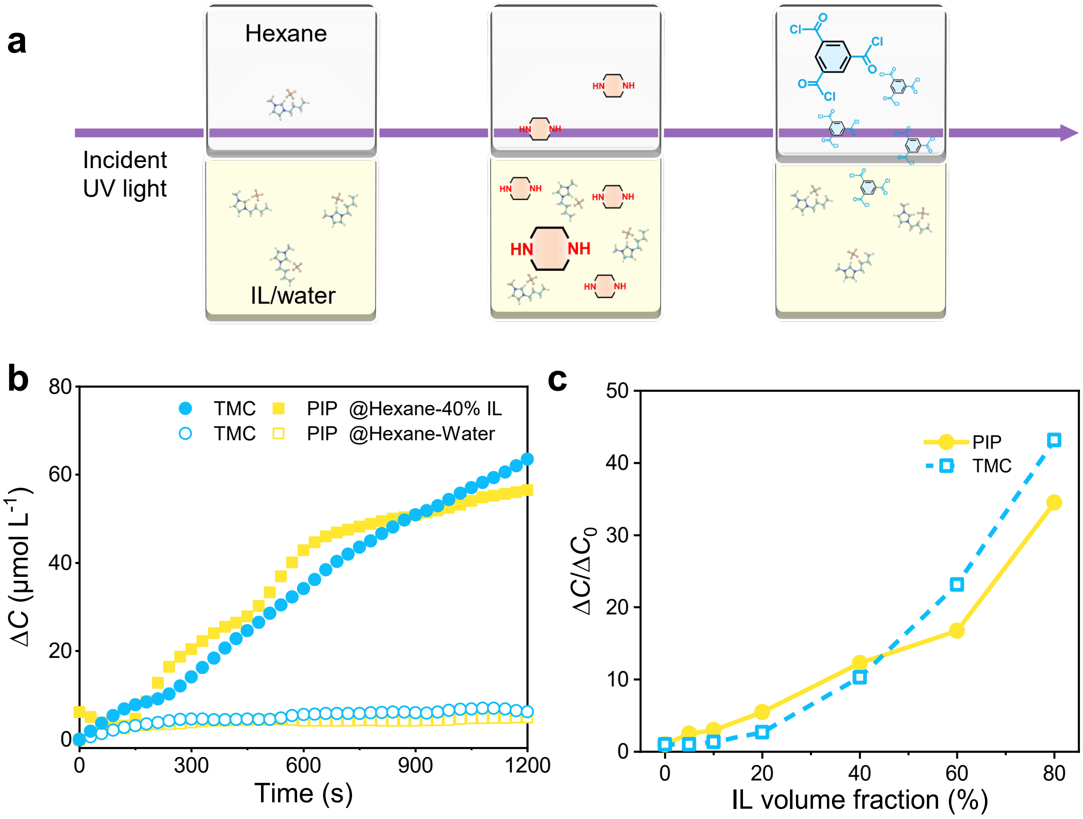

Supplementary Fig. 6 (a) Diagrams of in-situ monitoring of [Bmim][BF_4], PIP, and TMC, diffused across the interfaces between hexane and different IL/water mixtures. (b) Concentration variations of PIP and TMC versus time at the hexane-water or hexane-40 v/v% IL interface. (c) Plotted concentration changes (ΔC) of PIP and TMC during 1200 s with increasing IL content, where ΔC_0 is the monomer diffusion amount at the pristine hexane-water interface.

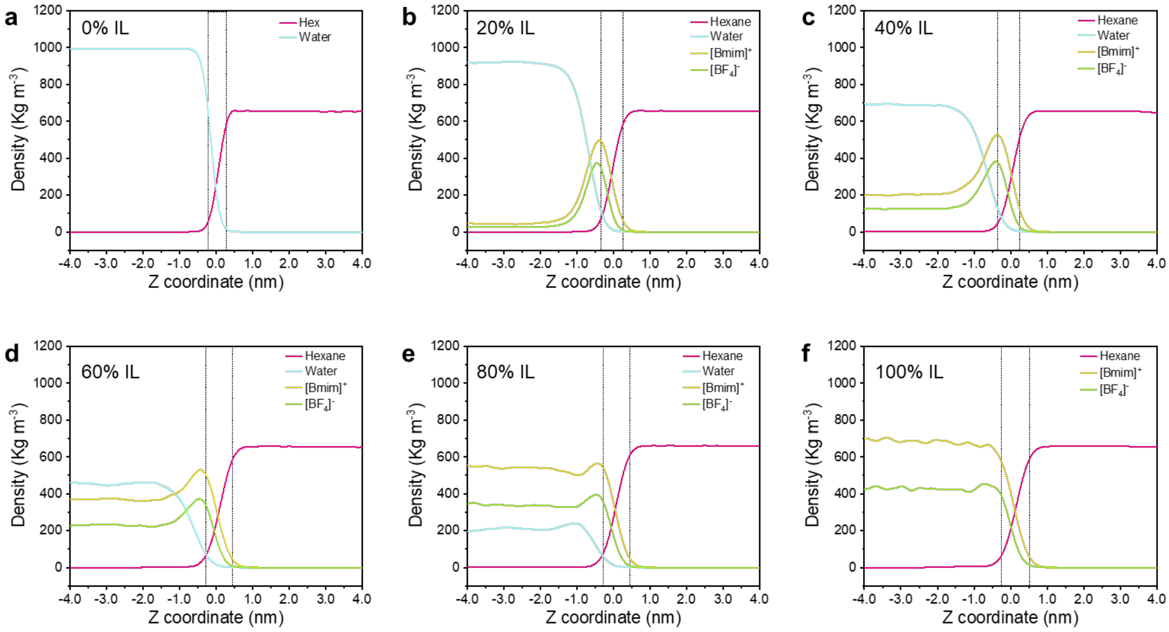

Supplementary Fig. 7 Density distributions perpendicular to the interface of hexane and IL/water mixture: The IL volume fractions are (a) 0 v/v%, (b) 20 v/v%, (c) 40 v/v%, (d) 60 v/v%, (e) 80 v/v%, and (f) 100 v/v%, respectively. These IL/water mixture, interface, and hexane regions are separated by the dashed lines.

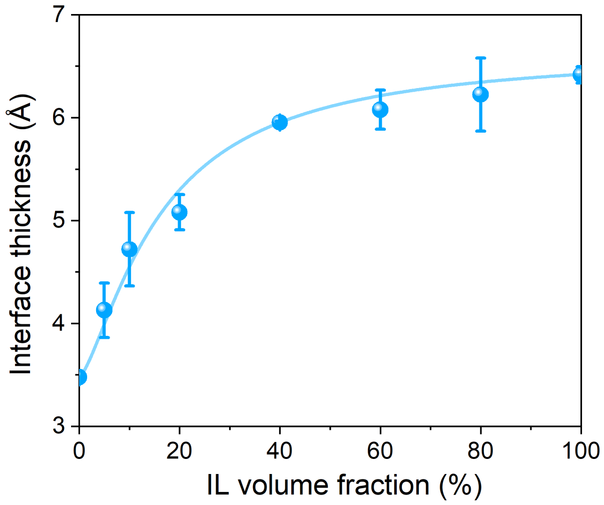

Supplementary Fig. 8 The thickness of the interfaces between hexane and IL/water mixture versus the IL volume fraction. The interface thickness is defined as the scope of 10 wt% - 90 wt% hexane from the density profile which is a common method to define interface position. The error bar is standard deviation of three replicate caliculations.

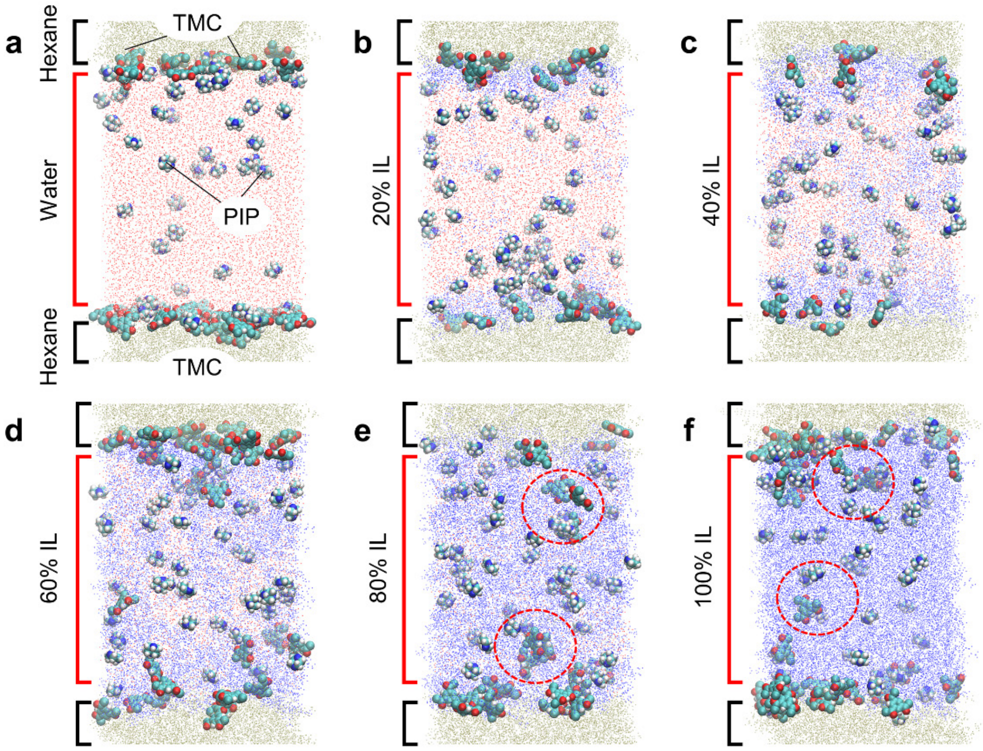

Supplementary Fig. 9 Snapshots of the simulated equilibrium distribution of PIP and TMC molecules between the hexane and different IL/water solutions. The IL volume fractions are (a) 0 v/v%，(b) 20 v/v%，(c) 40 v/v%，(d) 60 v/v%，(e) 80 v/v%，and (f) 100 v/v%, respectively. The red dashed circles refer to the TMC molecules distributed in the IL/water side.

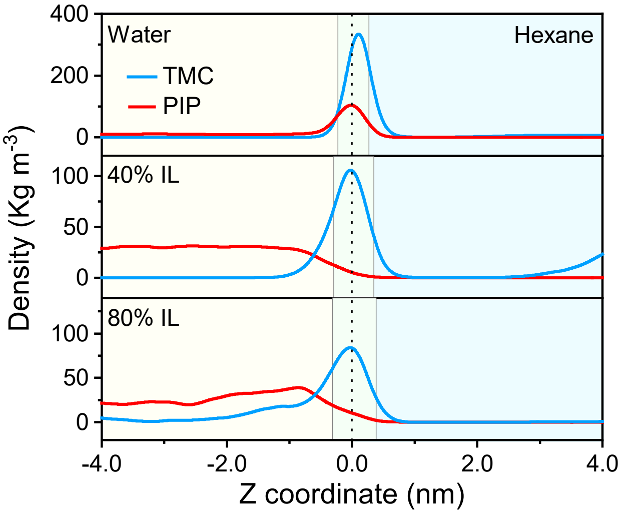

Supplementary Fig. 10 Simulated equilibrium density distribution of PIP and TMC molecules along the z direction perpendicular to the interface of hexane and IL/water mixture.

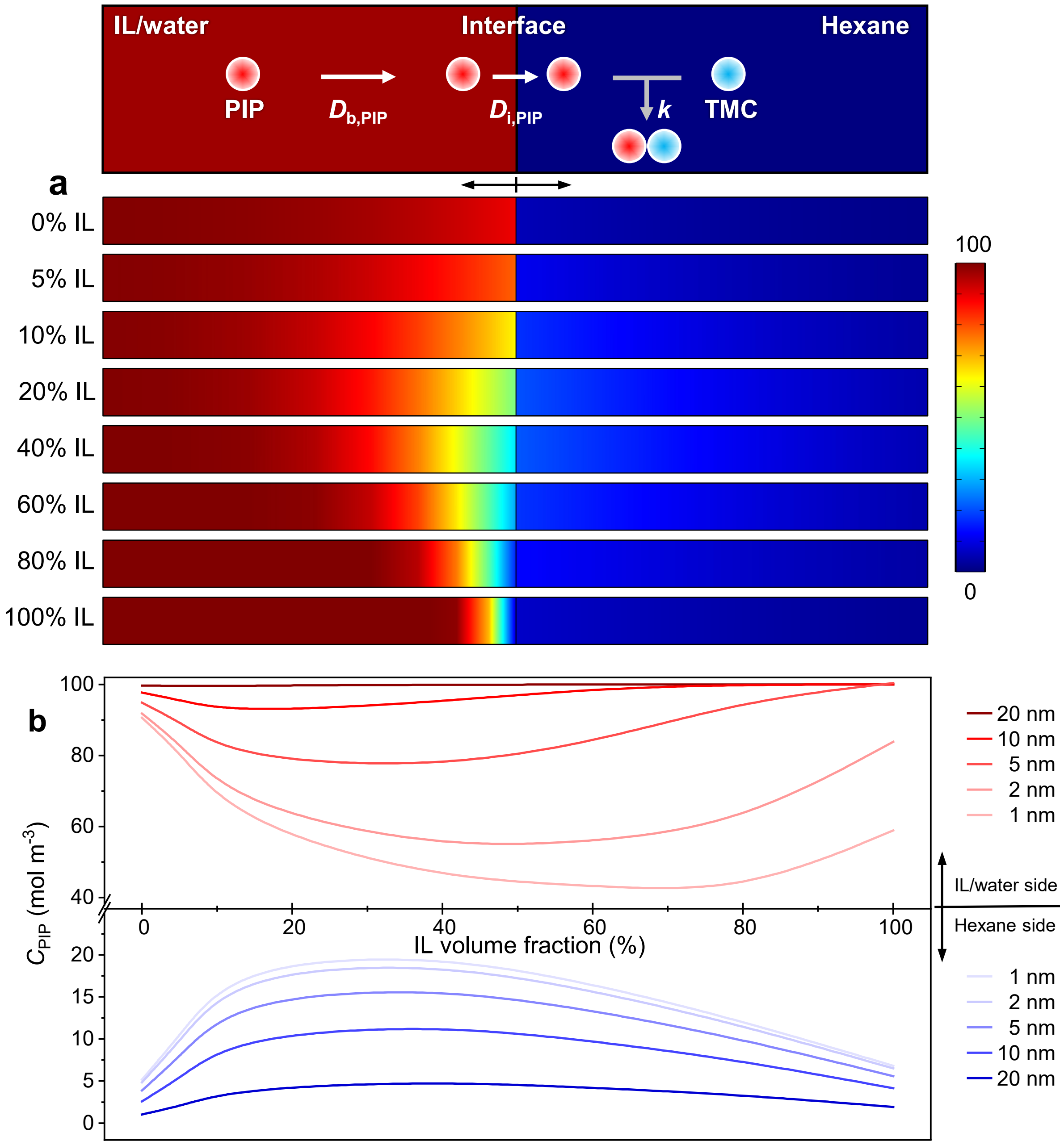

Supplementary Fig. 11 PIP distributions and diffusion amount (C_{PIP}) at the interface between hexane and IL/water mixture, calculated based on the quantified diffusion coefficients. (a) Schematic illustration of PIP diffusion behavior and the snapshots of PIP distribution in the 20 nm range around the interface at the time of 50 ns. The rainbow colorbar represents the PIP concentration. (b) PIP concentrations versus the IL volume fraction at different locations away from the interface of hexane and IL/water mixture.

The reaction rate of interfacial polymerization has a great impact on the polyamide growth and membrane properties. To delve deeper into the issue regarding the influence of IL addition on the reaction rate and the charge properties of polyamide membranes, we endeavored to formulate a simplified kinetic model for interfacial polymerization. This model concurrently considers the interfacial diffusion rate constant (k_d) of PIP and polymerization reaction rate constant (k_r), and hypothesizes the charge character of the polyamide membrane based on the relative concentrations of the two monomers within the reaction region. Initially, k_d of PIP was computed utilizing a method combining MD simulations and transition state theory, introduced within this work. Subsequently, k_r of PIP and TMC in n-hexane as a medium was investigated via DFT calculations (Supplementary Fig. 12).

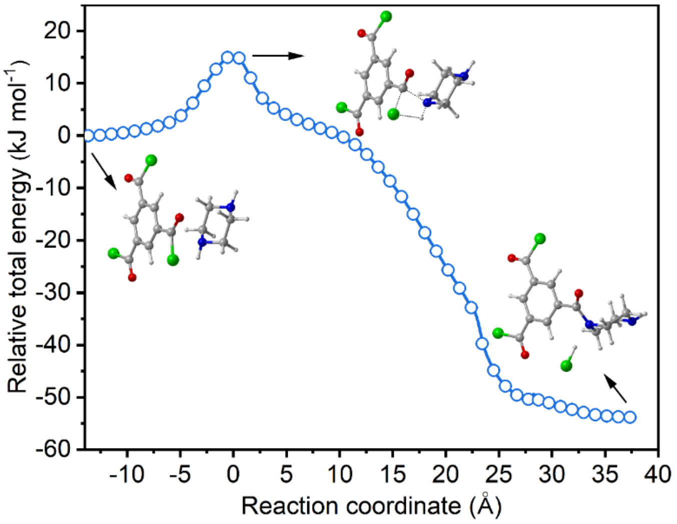

Supplementary Fig. 12 Total energy variation of the intrinsic reaction coordinate along the Schotten-Baumann reaction between PIP and TMC. The gray, white, blue, and red spheres represent carbon, hydrogen, nitrogen, and oxygen, respectively.

Given that the interfacial diffusion of PIP is governed by the concentration gradient and partition coefficient P, it follows a first-order kinetic process. We describe the temporal variation of concentrations of PIP, TMC, and synthesized amide in the reaction zone during the initial stage of interfacial polymerization with a set of ordinary differential equations:

$$\frac{dc_{\mathrm{amide}}}{dt} = k_r c_{\mathrm{PIP}} c_{\mathrm{TMC}} \qquad \text{(Supplementary equation 1)}$$

$$\frac{dc_{\mathrm{PIP}}}{dt} = \alpha P k_d \left(1 - \frac{c_{\mathrm{amide}}}{c_{\mathrm{amide}} + c_{\mathrm{TMC}}}\right)^{\beta} \left(c_{w\mathrm{PIP}} - c_{\mathrm{PIP}}\right) - k_r c_{\mathrm{PIP}} c_{\mathrm{TMC}} \qquad \text{(Supplementary equation 2)}$$

$$\frac{dc_{\mathrm{TMC}}}{dt} = -k_r c_{\mathrm{PIP}} c_{\mathrm{TMC}} \qquad \text{(Supplementary equation 3)}$$

where c_{amide}, c_{PIP}, $c_{w\mathrm{PIP}}$, and c_{TMC} denote the concentrations of the resulting amide, PIP in the reaction zone, initial PIP in solution, and TMC in the reaction zone, respectively. α represents a scaling factor, reflecting the supplementary rate difference of the bulk diffusion process to the initial interfacial diffusion concentration. According to Fick's diffusion law, α is directly proportional to the self-diffusion coefficient of PIP in the solution, estimated to be 1.00, 0.97, 0.82, 0.61, 0.39, 0.23, 0.08 and 0.02 for the IL proportions of 0 v/v%, 2 v/v%, 10 v/v%, 20 v/v%, 40 v/v%, 60 v/v%, 80 v/v%, 100 v/v%, respectively. The initial condition at $t = 0$ are $c_{\mathrm{amide}} = c_{\mathrm{PIP}} = 0$ and $c_{\mathrm{TMC}} = 0.009$ M. This model operates on the premise of equivalent activity of functional groups and disregards the Gibbs isothermal adsorption of monomers at the interface. Moreover, a scaling similar to that employed by Freger et al. is used to embody the self-limiting effect, with the scaling coefficient β set to 2 in this model[7]. The partition coefficient is derived from our previous study[8], here $P = 0.0016$. The computed k_r is 6.15×10^8 s^{-1} M^{-1}, and k_d values are listed in the Supplementary Table 4.

Our computational findings illustrate that, with a gradual increase in the IL addition, the concentration of PIP in the reaction zone initially increases and subsequently declines, occupying a superior position to TMC concentration when the IL volume fraction ranges from 20 v/v% to 40 v/v% (Supplementary Fig. 13). This outcome is consistent with the observed trends in membrane charge properties and the proposed mechanism. The intrinsic reasons for this variation may be distilled into three key points. Firstly, the elevation in the reaction rate is attributed to the surge in PIP concentration within the reaction zone, which, intriguingly, lags behind the elevation in diffusion rate, indicating that interfacial diffusion process can dominate the monomer concentration ratio within the reaction zone. Secondly, the interfacial diffusion rate of PIP and the polymerization reaction rate are proximate, suggesting that the self-limiting effect induced by polymerization can rapidly freeze the monomer ratio within the reaction zone, which is reflected in the unreacted groups of polyamides. Thirdly, due to the viscosity increases, bulk diffusion is suppressed at higher IL contents, leading to an attenuated overall interfacial diffusion rate. In summary, both the reaction and diffusion rates collaboratively steer the

monomer concentration distribution in the initial phase of interfacial polymerization, thereby influencing the end groups of polyamides, and ultimately modulating their charge characteristics.

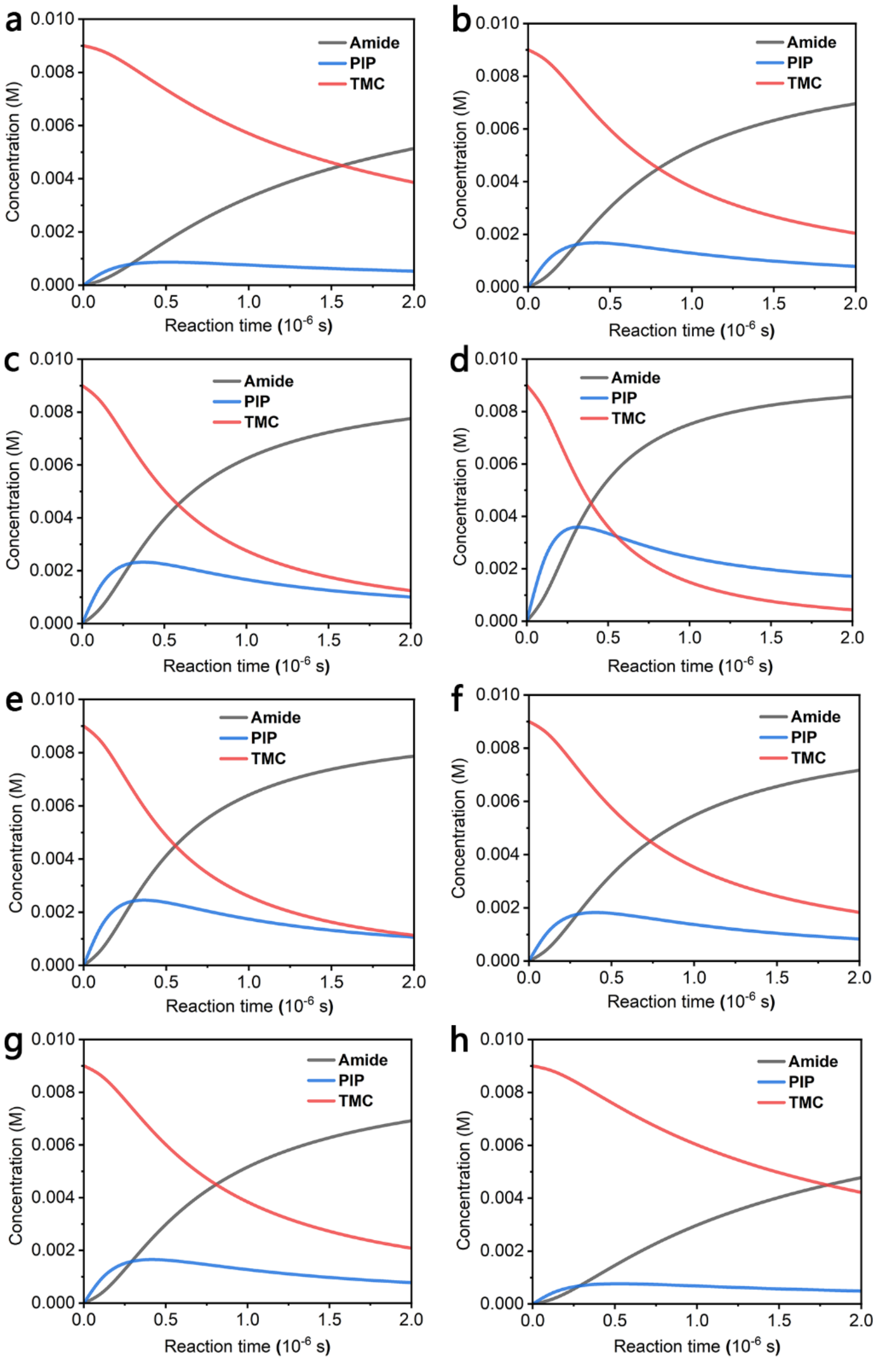

Supplementary Fig. 13 Concentration variation of species in reaction zone during the initial stage of interfacial polymerization, corresponding respectively to ionic liquid contents of (a) 0 v/v%, (b) 5 v/v%, (c)10 v/v%, (d) 20 v/v%, (e) 40 v/v%, (f) 60 v/v%, (g) 80 v/v%, and (h) 100 v/v%.

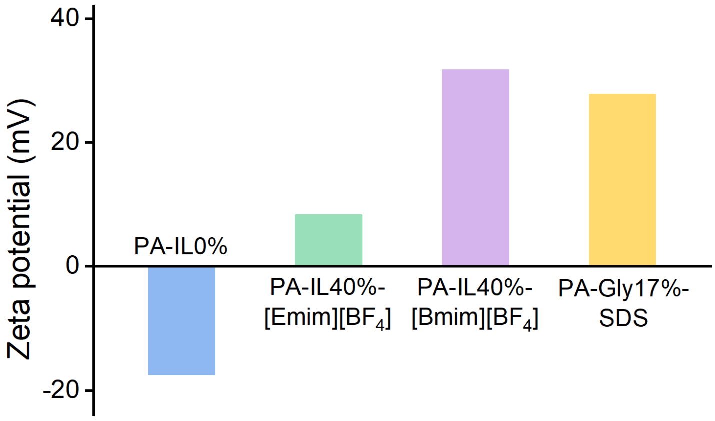

Supplementary Fig. 14 Comparison of the zeta potential at pH 6 between PA-IL0%, PA-IL40%-[Emim][BF_4], PA-IL40%-[Bmim][BF_4], and PA-Gly17%-SDS. The aqueous phase for the synthesis of PA-Gly17%-SDS comprised SDS at a concentration of 4.5 mM and glycerol at a volume fraction of 17 v/v%, with all other reaction conditions remaining consistent. This formulation was specifically designed to replicate the interfacial tension and viscosity characteristics of a 40 v/v% [Bmim][BF_4]/water solution.

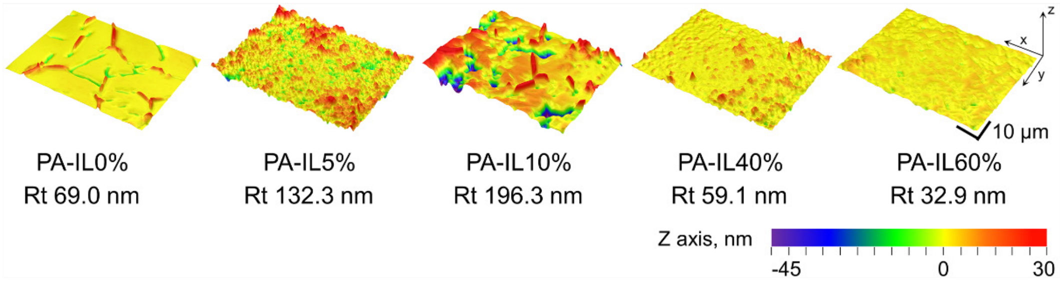

Supplementary Fig. 15 Surface morphology variation of polyamide nanofilms observed from macroscopic interferometric pictures. Area: 63×47 μm^2.

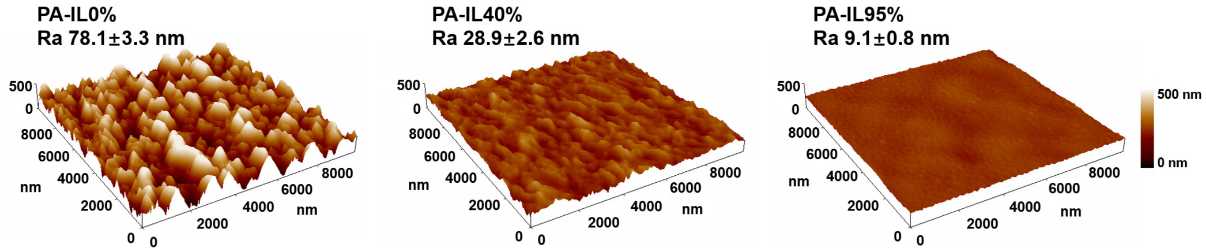

Supplementary Fig. 16 Surface morphology of PA-IL membranes characterized by AFM. Area: 10 μm × 10 μm.

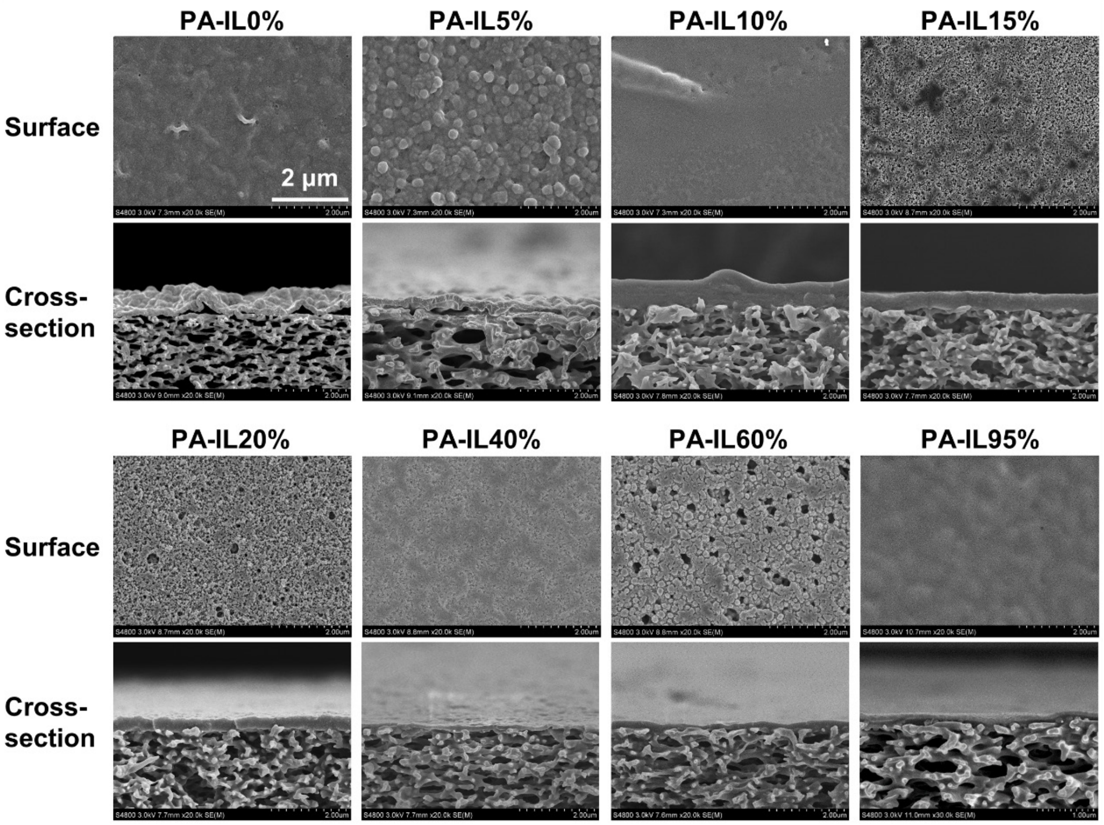

Supplementary Fig. 17 High-magnification SEM images of PA-IL membranes where polyamide nanofilms are supported by PES substrate.

AFM and SEM images present the nanoscale morphologies of polyamide nanofilms. The wrinkled polyamide surface gradually becomes smooth with increasing IL content because of a stable interface of alkane and viscous IL/water mixture. In the cross-sectional SEM images, polyamide nanofilms display a wave shape on the PA-IL0% membrane and then well fit the substrate with the IL content. The thickness of polyamide nanofilms rises to hundreds of nanometers first and subsequently declines.

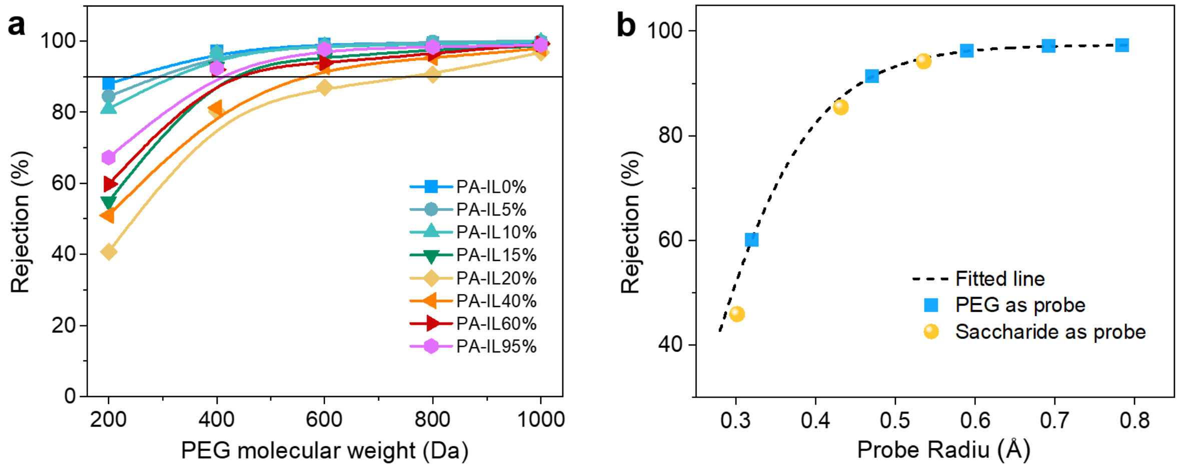

Supplementary Fig. 18 Rejection curves of PA-IL membranes evaluated by neutral organic PEG or saccharide probes. (a) PEG rejection curves of different PA-IL membranes. (b) Rejection curves of PA-IL40% membrane with neutral PEG or saccharide as probes. The x axis is the solute sizes (r) of PEG (Mw 200 Da, 400 Da, 600 Da, 800 Da, and 1000 Da) and saccharides (glucose, sucrose and raffinose)[9]. For PEG, $r = 1.673\times10^{-11}M^{0.557}$. For small saccharide, $\log r = -1.4962 + 0.4654\log M$.

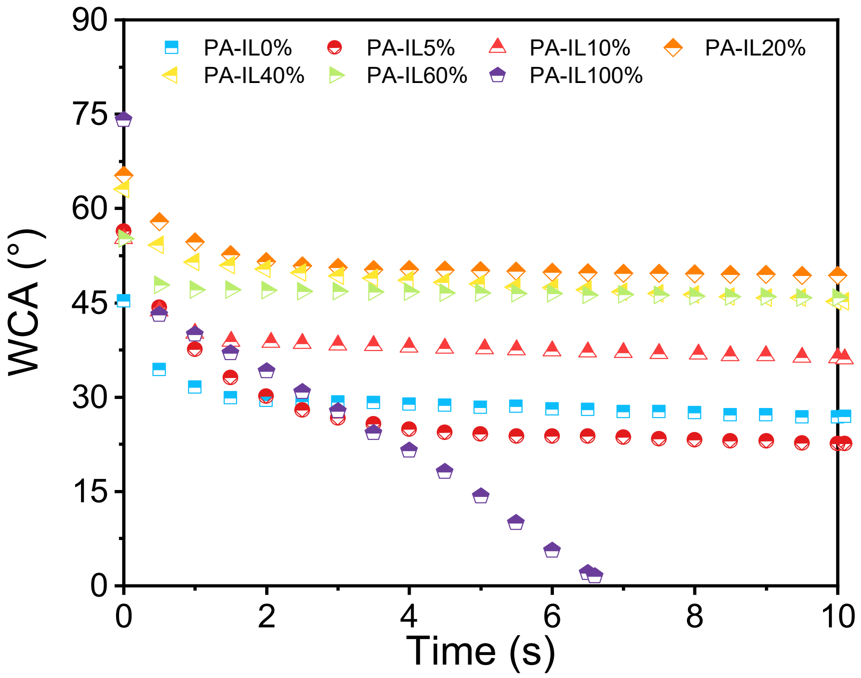

Supplementary Fig. 19 Water contact angles of PA-IL membranes.

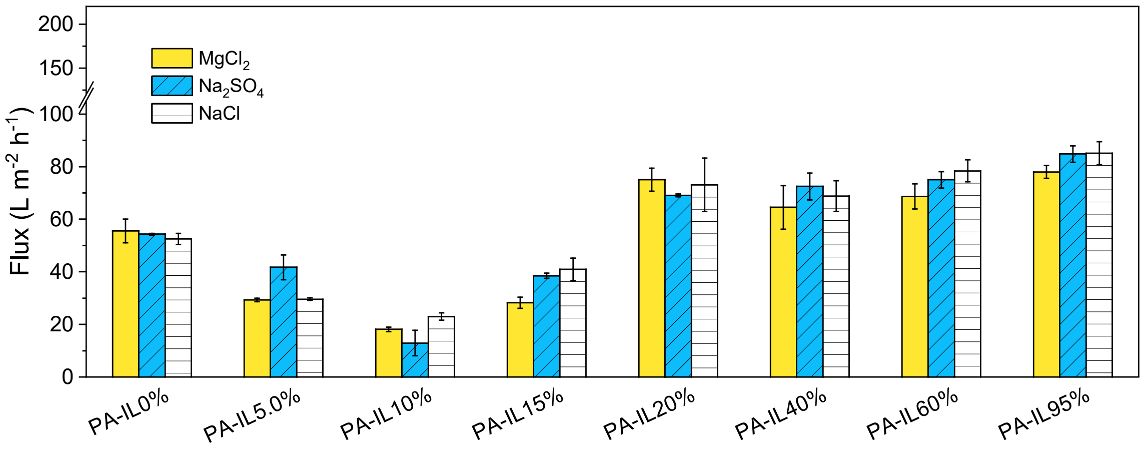

Supplementary Fig. 20 Water permeance of the PA-IL membranes. Feed concentration: 1.0 g L^{-1}. Data are presented as mean ± SD (n = 3).

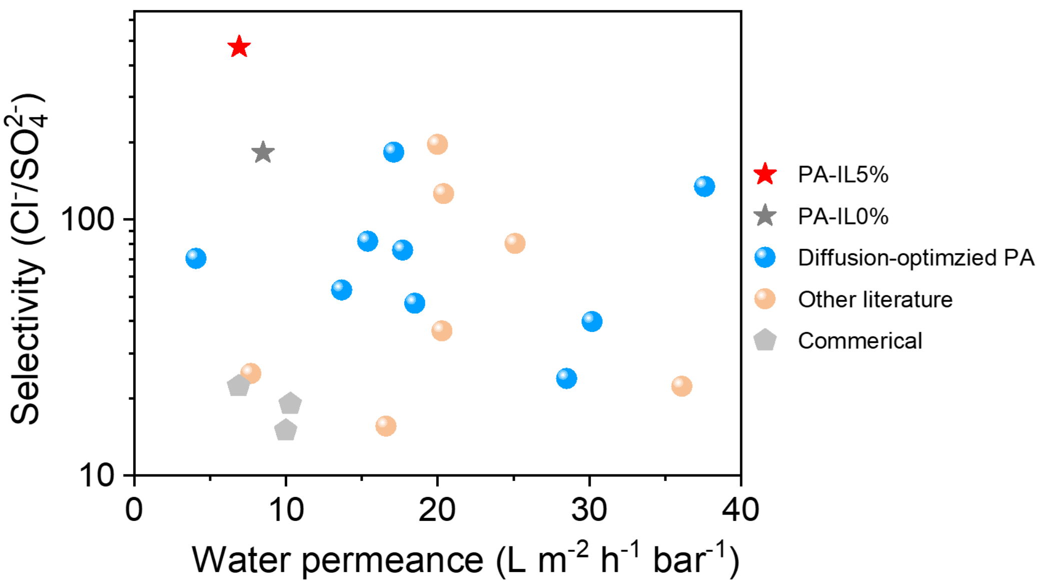

Supplementary Fig. 21 Performance comparison of the PA-IL5% and PA-IL0% membranes with the commercial and state-of-art PIP-based polyamide membranes prepared through diffusion-optimized interfacial polymerization or other novel manufacture methods. The detailed data are listed in in Supplementary Table 9.

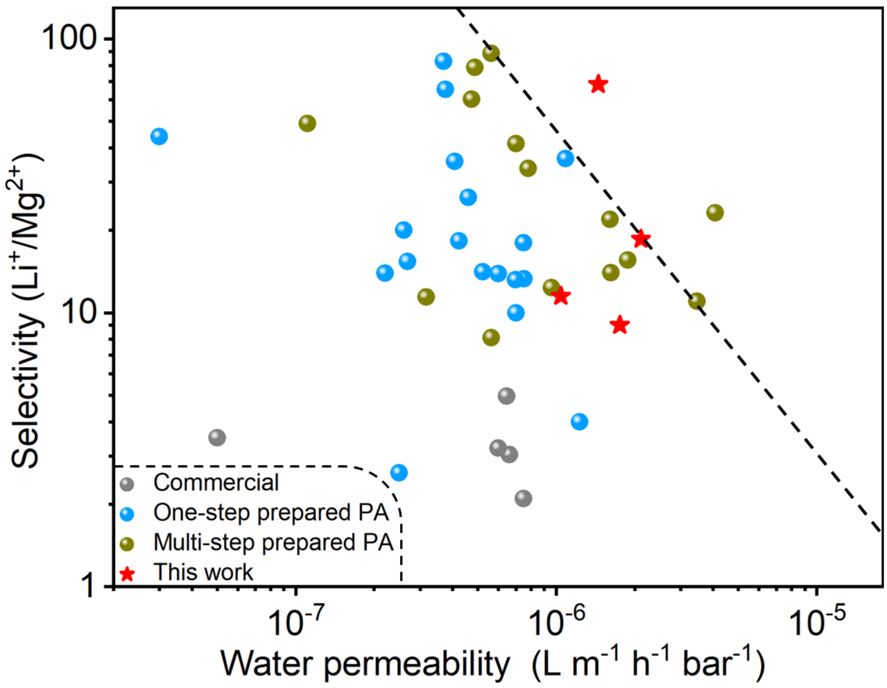

Supplementary Fig. 22 Comparison of the Li^+/Mg^{2+} selectivity and water permeability of PA-IL membranes with that state-of-art positively charged nanofiltration membranes. This includes membranes synthesized via one-step IP using PEI, novel monomers, or co-monomers, as well as membranes fabricated through multi-step IP featuring interlayer modification or post-grafting.

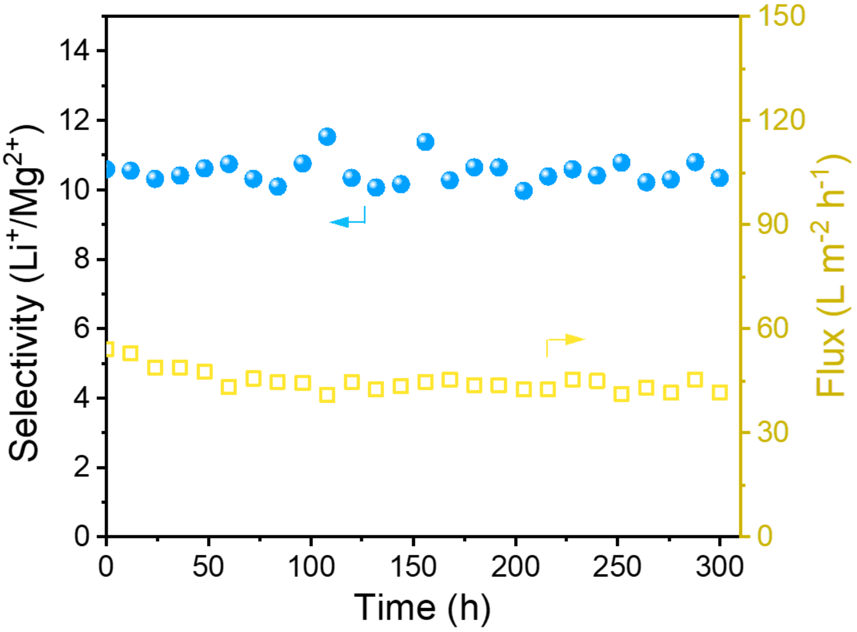

Supplementary Fig. 23 Long-term Li^{+}/Mg^{2+} separation performance of PA-IL40% membrane. Feed: 2.0 g L^{-1} $MgCl_2$ and 0.1 g L^{-1} LiCl at 6 bar.

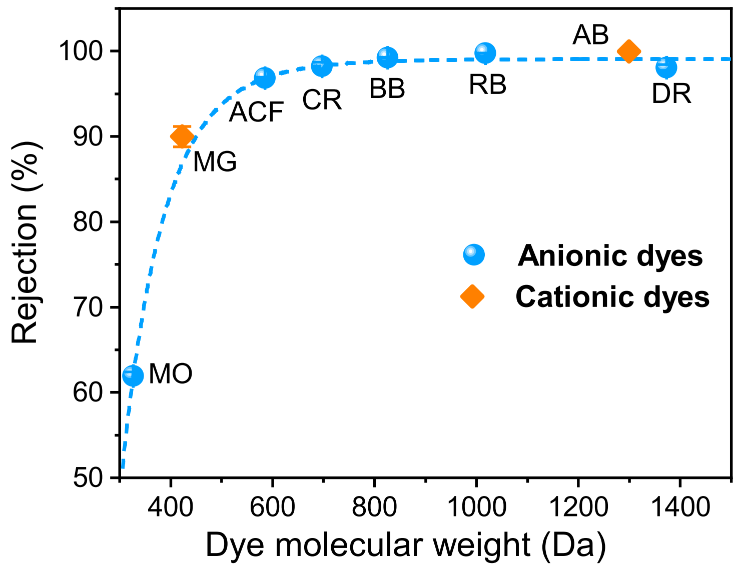

Supplementary Fig. 24 Rejection of PA-IL95% membrane for dyes of different molecular weights and charges. Feed solution: 0.1 g L^{-1}, 6 bar.

3. Supplementary Tables

Supplementary Table 1 Elemental content on the polyamide nanofilms of different PA-IL membranes from XPS analysis. IL refers to [Bmim][BF_4].

Samples*	*C* (%)	*N* (%)	*O* (%)	N/O
PA-IL0%	73.79	11.67	14.28	0.81
PA-IL5%	74.05	11.70	13.99	0.83
PA-IL10%	73.47	12.30	13.86	0.87
PA-IL20%	73.83	12.70	13.06	0.96
PA-IL40%	73.81	12.60	13.26	0.97
PA-IL60%	73.67	13.12	12.77	1.01
PA-IL100%	75.21	9.73	14.68	0.65

*All samples are washed thoroughly with DMF before characterization. Fluorine element (F) is too weak to be distinguished from the baseline noise within the range of 0.26%~0.43% lower than 0.5%.

Supplementary Table 2 XPS analysis of polyamide nanofilms on different PA-IL membranes. Binding energies, species, and their content ratios were determined from the high-resolution C1*s*, N1*s*, and O1*s* spectra.

**Samples**	**C1*s***		**N1*s***		**O1*s***	
	**Species**	**(%)**	**Species**	**(%)**	**Species**	**(%)**
PA-IL0%	C-H/C-C	54.32	R-N-H	3.22	N-C=O	80.39
	C-N	30.84	N-C=O	94.36		
	O-C=O/ N-C=O	14.84	R-N$^+$	2.42	O-C=O	19.61
PA-IL5%	C-H/C-C	57.23	R-N-H	5.04	N-C=O	78.75
	C-N	28.16	N-C=O	90.37		
	O-C=O/ N-C=O	14.31	R-N$^+$	4.6	O-C=O	21.25
PA-IL10%	C-H/C-C	56.49	R-N-H	6.05	N-C=O	80.04
	C-N	28.87	N-C=O	89.10		
	O-C=O/ N-C=O	14.65	R-N$^+$	4.85	O-C=O	19.96
PA-IL20%	C-H/C-C	56.67	R-N-H	7.82	N-C=O	81.43
	C-N	28.11	N-C=O	87.33		
	O-C=O/ N-C=O	15.22	R-N$^+$	4.85	O-C=O	18.57
PA-IL40%	C-H/C-C	54.75	R-N-H	10.61	N-C=O	81.61
	C-N	30.27	N-C=O	85.7		
	O-C=O/ N-C=O	14.38	R-N$^+$	3.69	O-C=O	18.39
PA-IL60%	C-H/C-C	55.65	R-N-H	11.54	N-C=O	80.09
	C-N	31.11	N-C=O	78.88		
	O-C=O/ N-C=O	13.24	R-N$^+$	9.58	O-C=O	19.91
PA-IL100%	C-H/C-C	61.90	R-N-H	4.05	N-C=O	78.94
	C-N	27.83	N-C=O	91.38		
	O-C=O/ N-C=O	10.27	R-N$^+$	4.57	O-C=O	21.06

Additional notes on membrane compactness

Generally, the PIP-TMC based polyamide membranes possess a complicated molecular structure comprising crosslinking structures (X), linear structures (Y) and two terminate structures with either amino (T_{amino}) or carboxyl groups (T_{carboxyl}), as depicted in Supplementary Fig. 25.

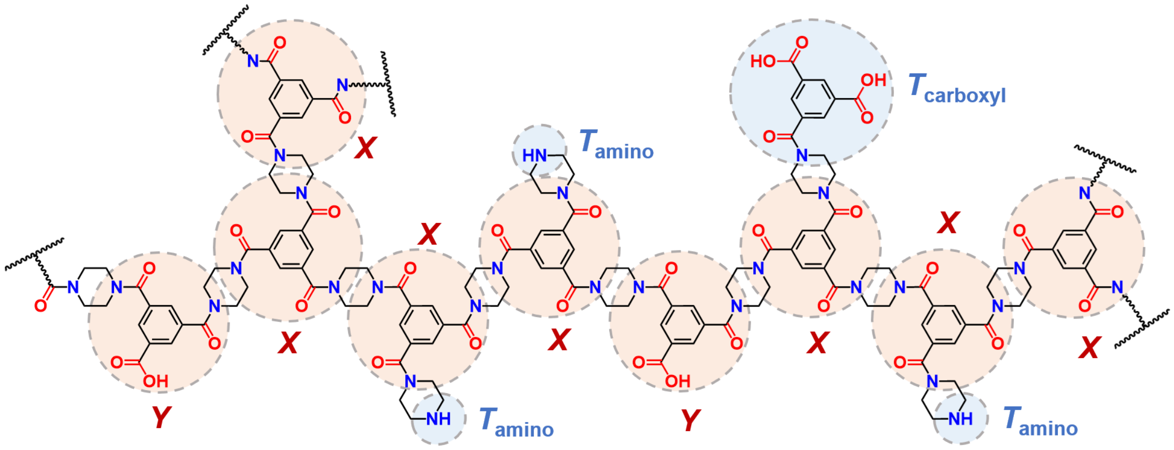

Supplementary Fig. 25 Schematic representation of all potential chemical structures in PIP-TMC polyamide membrane. Here, X, Y, T_{amino}, and T_{carboxyl} represent the crosslinked structure, linear structure, amino-terminated structure, and carboxyl-terminated structure, respectively.

Conventionally, the calculation of the degree of network crosslinking (DNC) includes only the crosslinking structures (X) and linear structures (Y), yet neglecting the terminal structures of T_{amino} or T_{carboxyl}:

$$DNC = \frac{X}{X+Y} \quad \text{(Supplementary equation 4)}$$

Given that each X consists of three O atoms and three N atoms, and each Y has four O atoms and two N atoms, the O/N ratio can be expressed by the following equation:

$$\frac{O}{N} = \frac{3X+4Y}{3X+2Y} \quad \text{(Supplementary equation 5)}$$

Consequently, DNC can be further expressed as:

$$DNC = \frac{4-2\frac{O}{N}}{1+\frac{O}{N}} \quad \text{(Supplementary equation 6)}$$

As a consequent, the membrane crosslinking degree is evaluated using the O/N ratio on the basis of Supplementary equation 6. However, owing to the fact that each T_{amino} contributes to one N atom and each T_{carboxyl} adds four O atoms, these N and O atoms have no contribution to crosslinking degree. Consequently, employing the total N and O atoms ratio from X-ray photoelectron spectroscopy (XPS) for *DNC* calculation lacks precision. This assertion is corroborated by several studies where *DNC* values of polyamide membranes are negative but still exhibit good separation performance[10-12].

To provide an accurate representation of the compactness of polyamide membranes, especially considering terminal groups, we introduce the concept of the amide group ratio ($r_{\mathrm{N\text{-}C=O}}$). This ratio is defined as the number of amide groups within the polyamide membrane relative to the total number of amide (n_{amide}), amino (n_{amino}), and carboxyl (n_{amino}) groups (Supplementary Fig. 26), providing a precise metric for assessing crosslinking density.

$$r_{\mathrm{N\text{-}C=O}} = \frac{n_{\mathrm{amide}}}{n_{\mathrm{amide}} + n_{\mathrm{amino}} + n_{\mathrm{carboxyl}}} \qquad \text{(Supplementary equation 7)}$$

From this equation, we found that a higher $r_{\mathrm{N\text{-}C=O}}$ means more formation of amide groups, implying a higher crosslinking degree and increased membrane compactness.

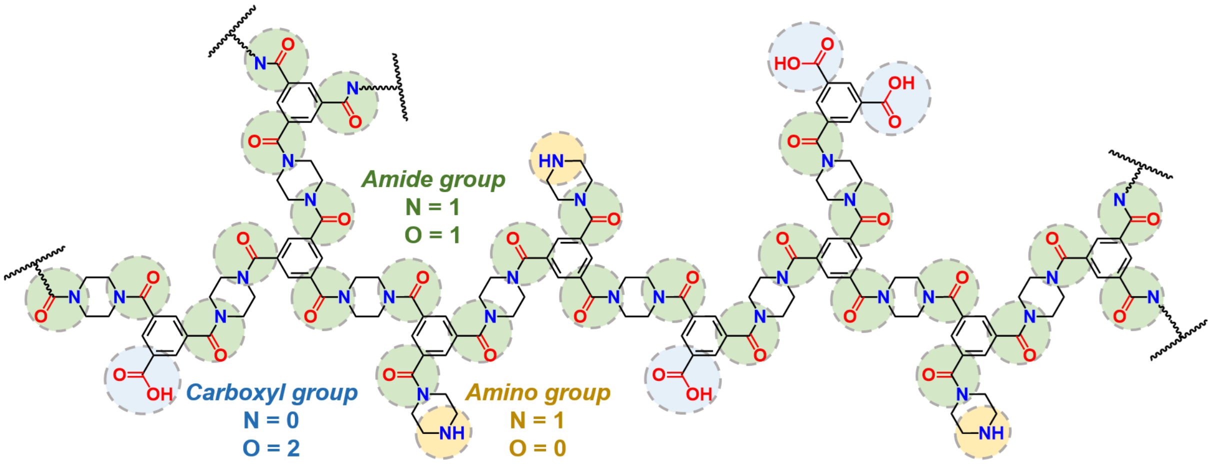

Supplementary Fig. 26 Schematic representation of all potential functional groups in polyamide and their corresponding N and O atom counts.

The quantification for n_{amide}, n_{amino}, and n_{carboxyl} is derived from XPS data, following the deconvolution of narrow N1*s* and O1*s* spectra. Specifically, n_{amide} is determined by the $N_{\text{(N-C=O)}}$:

$$n_{\text{amide}} = N_{\text{(N-C=O)}} \qquad \text{(Supplementary equation 8)}$$

while the sum of n_{amide} and n_{amino} is reflected in the total nitrogen content (N):

$$n_{\text{amide}} + n_{\text{amino}} = N \qquad \text{(Supplementary equation 9)}$$

n_{carboxyl} is calculated from the total oxygen content (O) minus the oxygen associated with N-C=O bonds ($O_{\text{(N-C=O)}}$):

$$n_{\text{carboxyl}} = \frac{O - O_{(N\text{-}C=O)}}{2} \qquad \text{(Supplementary equation 10)}$$

Thus, $r_{\text{N-C=O}}$ can be calculated from the following equation:

$$r_{\text{N-C=O}} = \frac{N_{\text{(N-C=O)}}}{N + \frac{O - O_{\text{(N-C=O)}}}{2}} = \frac{2N_{\text{(N-C=O)}}}{2N + O - O_{\text{(N-C=O)}}} \qquad \text{(Supplementary equation 11)}$$

Employing this method, we confirmed that the proportion of surface and internal amide bonds in the PA-IL40% membrane is approximately 80.3% and 70.9%, respectively (Supplementary Table 3). These values align with those in the PA-IL0% membrane (about 76%) known for its high crosslinking density. These results demonstrate that the PA-IL40% membrane possesses a robustly crosslinked structure.

Supplementary Table 3 Element profile and the deconvoluted amount of N-C=O groups in the inner PA-IL40% membrane.

**Etch time (s)**	***N* (%)**	***O* (%)**	**N/O ratio**	**$O_{\text{(N-C=O)}}$ (%)**	**$r_{\text{N-C=O}}$ (%)**
0	12.65	13.42	0.94	11.10	80.3
20	14.18	12.65	1.12	11.02	77.2
40	14.58	11.67	1.25	10.21	69.2
60	14.30	11.44	1.25	10.05	68.1
80	14.17	11.49	1.23	10.13	71.6
100	14.09	11.33	1.24	10.15	70.9

* N and O represent the total content of nitrogen and oxygen elements in the polyamide, respectively. $O_{(N\text{-}C=O)}$ denote the oxygen content within the amide groups. $r_{N\text{-}C=O}$ refers to the proportion of amide groups relative to the total functional groups present in the polyamide.

Supplementary Table 4 Molecular dynamics simulation results for different systems containing hexane and different IL/water mixtures, including the interface thickness, diffusion energy barrier (ΔG), interfacial diffusion rate constant (k_d), theoretical interfacial PIP diffusion rates (D_i) and bulk PIP diffusion rates (D_b).

**IL volume fraction (v/v%)**	**Interface thickness (Å)**	**ΔG (kJ mol^{-1})**	**$k_d \times 10^{-8}$ (s^{-1})**	**$D_i \times 10^7$ (cm^2 s^{-1})**	**$D_b \times 10^6$ (cm^2 s^{-1})**
0	3.5	29.3	0.4	0.55	9.3
5	4.1	27.7	0.9	1.48	9.0
10	4.7	26.2	1.6	3.55	7.6
20	5.1	25.0	3.8	10.0	5.7
40	5.9	24.7	3.6	12.7	3.6
60	6.1	23.8	4.2	15.5	2.1
80	6.2	21.5	10.7	41.1	0.79
100	6.4	20.3	17.3	70.9	0.21

Supplementary Table 5 Surface tension and viscosity of a series of IL/water solutions. Data are presented as mean ± SD (n = 3).

**IL volume fraction (v/v%)**	**Surface tension (mN m^{-1})**	**Viscosity (mPa·s)**
0	72.7 ± 0.2	1.04 ± 0.03
2.5	61.3 ± 0.9	--
5.0	56.1 ± 0.4	--
10	50.8 ± 0.1	1.23 ± 0.03
15	48.3 ± 0.3	--
20	47.2 ± 0.2	1.49 ± 0.05
40	46.4 ± 0.1	2.28 ± 0.05
60	46.3 ± 0.1	3.67 ± 0.02
80	46.0 ± 0.1	7.64 ± 0.07
100	44.8 ± 0.4	107.9 ± 0.6

Supplementary Table 6 Zeta potential, polyamide thickness, molecular weight cut-off (MWCO), and pore distribution of PA-IL membranes. μ_p is the estimated mean pore size, and σ_p is the geometric pore size deviation. The polyamide thickness value is presented as mean ± SD (n = 3).

**Membranes**	**Zeta potential (mV)**	**Thickness (nm)**	**MWCO (Da)**	**μ_p (nm)**	**σ_p**
PA-IL0%	-17.5	130 ± 18	212	0.32	1.74
PA-IL5%	-15.2	334 ± 34	256	0.34	1.91
PA-IL10%	7.6	483 ± 32	276	0.44	1.55
PA-IL15%	10.1	336 ± 38	441	0.56	1.56
PA-IL20%	19.3	219 ± 13	669	0.69	1.59
PA-IL40%	34.0	132 ± 14	550	0.63	1.57
PA-IL60%	10.0	180 ± 52	438	0.57	1.57
PA-IL80%	1.45	--	> 1000	--	--
PA-IL95%	0.95	70 ± 8	378	0.50	1.60
PA-IL100%	-5.22	--	> 1000	--	--

Supplementary Table 7 Nanofiltration performance of PA-IL membranes at 6 bar. Concentration of feed solution: 1.0 g L^{-1}. Data are presented as mean ± SD (n = 3).

	**Na_2SO_4**		**$MgCl_2$**		**NaCl**		**LiCl**	
	**Flux (LMH)**	**R (%)**	**Flux (LMH)**	**R (%)**	**Flux (LMH)**	**R (%)**	**Flux (LMH)**	**R (%)**
PA-IL0%	54.3 ± 0.3	98.5 ± 0.5	55.5 ± 4.5	89.3 ± 3.5	52.5 ± 2.1	54.1 ± 5.9	51.1 ± 0.7	32.2 ± 0.4
PA-IL5%	41.7 ± 4.7	98.1 ± 0.9	29.3 ± 0.7	89.2 ± 0.3	29.6 ± 0.5	36.9 ± 1.0	33.8 ± 5.7	29.8 ± 0.9
PA-IL10%	12.9 ± 4.8	47.7 ± 12.3	18.1 ± 0.9	98.2 ± 0.2	23.0 ± 1.4	69.6 ± 0.4	21.4 ± 1.9	51.1 ± 2.4
PA-IL15%	38.5 ± 1.0	38.2 ± 2.6	28.2 ± 2.1	96.8 ± 0.2	40.9 ± 4.3	65.0 ± 3.1	34.2 ± 0.6	65.1 ± 3.6
PA-IL20%	69.1 ± 0.5	40.6 ± 3.8	75.0 ± 4.4	93.8 ± 0.3	73.1 ± 10.1	52.9 ± 1.2	76.9 ± 10.7	43.0 ± 2.9
PA-IL40%	82.5 ± 5.1	31.6 ± 1.0	64.5 ± 8.3	95.1 ± 0.2	68.8 ± 5.8	59.9 ± 3.6	70.1 ± 10.3	52.9 ± 0.6
PA-IL60%	75.0 ± 3.1	25.8 ± 0.4	68.7 ± 4.8	96.3 ±1.3	78.4 ± 4.2	46.7 ± 1.7	77.8 ± 4.1	51.5 ± 1.2
PA-IL95%	84.8 ± 3.1	15.5 ± 0.5	78.0 ± 2.5	45.5 ± 0.3	85.1 ± 4.4	11.4 ± 1.9	--	--

Supplementary Table 8 Separation performance of negative PA-IL membranes for mixed solution containing 500 ppm Na_2SO_4 and 500 ppm NaCl.

**Membranes**		**R(SO_4^{2-})**	**R(Cl^-)**	**S(Cl^-/SO_4^{2-})**	**Reference**
PA-IL0%	1	99.68%	31.4%	213	This work
	2	99.65%	20.1%	226	
	3	99.65%	36.4%	182	
PA-IL5%	1	99.81%	17.5%	430	
	2	99.83%	15.7%	483	
	3	99.86%	17.5%	585	
SCOF/PA		99.6%	--	312.6	[13]
ULPA-2		99.86%	8.1%	656.2	[14]

Supplementary Table 9 Performance comparison of PA-IL membranes with state-of-art membranes in Cl^-/SO_4^{2-} separation.

Membranes	Permeance ($L\ m^{-2}\ h^{-1}\ bar^{-1}$)	Separation factor (Cl^-/SO_4^{2-})	Reference
**This work**			
PA-IL0%	8.5	182	--
PA-IL5%	6.9	470	--
**Commercial membranes**			
NF-270	10.3	19	15
NF-90	6.7	5	16
NTR-7450	10.9	5.9	17
DL-GE Osmonics	10.0	15.0	18
HL-GE Osmonics	6.9	22.3	
**State-of-art PIP-based polyamide membranes**			
PA	4.1	70.4	19
Co_7:3@PA	13.7	52.9	
M-TB1-P20	18.5	47.0	20
PVP-PIP-TMC	30.2	40.0	21
NF3	28.5	23.9	22
PAN-PCA-PA	17.7	75.9	23
PDA-PA-PSF6	15.4	82.1	24
SDS-PIP-TMC	17.1	182.5	25
SLS-PIP-TMC3	37.6	134.4	26
TFC-spinIP	36.1	22.3	
TFC-IP	7.7	25	27
TFC-spinIP	20.3	36.7	
Free-IP	25.1	80.6	28
Rod coated-PA	20.4	125.7	29
Vaccum/PIP-TMC	20.0	196	30
Electrospray/Pip-TMC	16.6	15.5	31

Supplementary Table 10 Performance comparison of various membranes in Li^{+}/Mg^{2+} separation.

Membranes	Permeability ($L\ m^{-1}\ h^{-1}\ bar^{-1}$)	Separation factor (Li^{+}/Mg^{2+})	Reference
**This work**			
PA-IL10%	1.45×10^{-6}	68	--
PA-IL15%	2.12×10^{-6}	19	--
PA-IL20%	1.75×10^{-6}	9	--
PA-IL40%	1.04×10^{-6}	12	--
**One-step prepared polyamide membranes**			
NF270	6.63×10^{-7}	2.5	[32]
NF90	7.50×10^{-7}	5.02	[33]
DL	5.00×10^{-8}	3.5	[34]
DK	6.00×10^{-7}	3.2	
BAPP-TMC	2.50×10^{-7}	2.6	[35]
AEP/TMC=15	1.23×10^{-6}	4	[36]
AEP/TMC=240	7.00×10^{-7}	10	
PPA	3.76×10^{-7}	65.5	[36]
PEI-TMC	7.53×10^{-7}	13.3	[37]
PEI-TMC	2.50×10^{-7}	13.93	[38]
PEI-TMC	2.60×10^{-7}	20	[39]
PEI/POSS-TMC	6.00×10^{-7}	13.9	[40]
PEI/PIP-TMC	4.24×10^{-7}	18.3	[41]
HACC/PIP-TMC	6.28×10^{-7}	115	[42]
PIP-TMC/AB2	4.08×10^{-7}	35.7	[43]
PIP/PHF-TMC	6.99×10^{-7}	13.2	[44]
MCPM-2.0	7.50×10^{-7}	18	[45]
PAA-TMC	3.70×10^{-7}	82.8	[39]
TG-TMC	3.00×10^{-8}	43.9	[46]
N-CPTC-TAEA	1.09×10^{-6}	36.5	[47]
PHMG-TMC	5.24×10^{-7}	14.1	[48]
PHMB-TMC	4.61×10^{-7}	26.4	
**Multi-step prepared polyamide membranes**			
DETA-g-PIP-TMC	3.47×10^{-6}	11	[49]
SP-PEI	9.60×10^{-7}	12.37	[50]
PEI-g-PIP-TMC	7.81×10^{-7}	33.6	[51]
PIP-TMC/[MimAP][TF2N]	5.64×10^{-7}	8.12	[52]
DETA-PEI-TMC	3.17×10^{-7}	11.4	[53]

QEDTP-PEI-TMC	1.88×10^{-6}	15.6	54
TQAIL-PEI-TMC	4.07×10^{-6}	23.2	55
QBPD-PEI-TMC	1.62×10^{-6}	6.2	56
DHTAB-PEI-TMC	4.84×10^{-7}	60.1	57
Noria/PEI/SDS-TMC	1.61×10^{-6}	21.9	58
Uio-66-NH_2PSF-PIP-TMC	4.87×10^{-7}	78.6	59
NoriaPG/PEI interlayer/PIP-TMC	5.63×10^{-7}	88.6	60
cCOF interlayer/PEI-TMC	1.11×10^{-7}	49.1	61
i-PPA-a	7.00×10^{-7}	41.4	36

Supplementary References

1. M. J. Frisch, G. W. Trucks, H. B. Schlegel, G. E. Scuseria, M. A. Robb, J. R. Cheeseman, G. Scalmani, V. Barone, G. A. Petersson, H. Nakatsuji, X. Li, M. Caricato, A. V. Marenich, J. Bloino, B. G. Janesko, R. Gomperts, B. Mennucci, H. P. Hratchian, J. V. Ortiz, A. F. Izmaylov, J. L. Sonnenberg, Williams, F. Ding, F. Lipparini, F. Egidi, J. Goings, B. Peng, A. Petrone, T. Henderson, D. Ranasinghe, V. G. Zakrzewski, J. Gao, N. Rega, G. Zheng, W. Liang, M. Hada, M. Ehara, K. Toyota, R. Fukuda, J. Hasegawa, M. Ishida, T. Nakajima, Y. Honda, O. Kitao, H. Nakai, T. Vreven, K. Throssell, J. A. Montgomery Jr., J. E. Peralta, F. Ogliaro, M. J. Bearpark, J. J. Heyd, E. N. Brothers, K. N. Kudin, V. N. Staroverov, T. A. Keith, R. Kobayashi, J. Normand, K. Raghavachari, A. P. Rendell, J. C. Burant, S. S. Iyengar, J. Tomasi, M. Cossi, J. M. Millam, M. Klene, C. Adamo, R. Cammi, J. W. Ochterski, R. L. Martin, K. Morokuma, O. Farkas, J. B. Foresman, D. J. Fox, *Wallingford, CT* (2016).
2. Grimme, S., Antony, J., Ehrlich, S. & Krieg, H. A consistent and accurate ab initio parametrization of density functional dispersion correction (DFT-D) for the 94 elements H-Pu. *J. Chem. Phys.* **132**, 154104 (2010).
3. Grimme, S., Ehrlich, S. & Goerigk, L. Effect of the damping function in dispersion corrected density functional theory. *J. Comput. Chem.* **32**, 1456–1465 (2011).
4. Weigend, F. & Ahlrichs, R. Balanced basis sets of split valence, triple zeta valence and quadruple zeta valence quality for H to Rn: Design and assessment of accuracy. *Phys. Chem. Chem. Phys.* **7**, 3297–3305 (2005).
5. Lu, T. Tstcalculator. *Http://Sobereva.Com/310,* Accessed 12th Oct (2023).
6. Skodje, R. T. & Truhlar, D. G. Parabolic tunneling calculations. *J. Phys. Chem.* **85**, 624–628 (1981).
7. Freger, V. Kinetics of film formation by interfacial polycondensation. *Langmuir* **21**, 1884–1894 (2005).
8. Liu, C. *et al.* Interfacial polymerization at the alkane/ionic liquid interface. *Angew. Chem. Int. Ed.* **60**, 14636–14643 (2021).
9. Zhang, S., Fu, F. & Chung, T.-S. Substrate modifications and alcohol treatment on thin film composite membranes for osmotic power. *Chem. Eng. Sci.* **87**, 40–50 (2013).
10. Tian, B. et al. Nanofiltration membrane combining environmental-friendly polycarboxylic interlayer prepared from catechol for enhanced desalination performance. *Desalination* **512**, 115118 (2021).
11. Jiang, C. et al. Ultrathin film composite membranes fabricated by novel in situ free interfacial polymerization for desalination. *ACS Appl. Mater. Interfaces* **12**, 25304–25315 (2020).
12. Yuan, S. *et al.* Nanofiltration membranes with cellulose nanocrystals as an interlayer for unprecedented performance. *J. Mater. Chem. A* **8**, 3238–3245 (2020).
13. Xu, S. *et al.* Anionic covalent organic framework as an interlayer to fabricate negatively charged polyamide composite nanofiltration membrane featuring ions sieving. *Chem. Eng. J.* **427**, 132009 (2022).

14. Li, Y. *et al.* Graphene quantum dot engineered ultrathin loose polyamide nanofilms for high-performance nanofiltration. *J. Mater. Chem. A* **8**, 23930–23938 (2020).
15. Li, Y., Wong, E., Mai, Z. & Van der Bruggen, B. Fabrication of composite polyamide/Kevlar aramid nanofiber nanofiltration membranes with high permselectivity in water desalination. *J. Membr. Sci.* **592**, 117396 (2019).
16. Fang, W., Shi, L. & Wang, R. Mixed polyamide-based composite nanofiltration hollow fiber membranes with improved low-pressure water softening capability. *J. Membr. Sci.* **468**, 52–61 (2014).
17. Schaep, J., Van Der Bruggen, B., Vandecasteele, C. & Wilms, D. Influence of ion size and charge in nanofiltration. *Sep. Purif. Technol.* **14**, 155–162 (1998).
18. Tang, Y.-J., Xu, Z.-L., Xue, S.-M., Wei, Y.-M. & Yang, H. A chlorine-tolerant nanofiltration membrane prepared by the mixed diamine monomers of PIP and BHTTM. *J. Membr. Sci.* **498**, 374–384 (2016).
19. Lin, Y. *et al.* Zwitterionic copolymer-regulated interfacial polymerization for highly permselective nanofiltration membrane. *Nano Lett.* **21**, 6525–6532 (2021).
20. Liu, S. *et al.* Tröger's base-regulated interfacial polymerization of polyamide nanofiltration membranes with enhanced performance. *J. Membr. Sci.* **682**, 121787 (2023).
21. Li, Z. *et al.* Innovative role of polyvinylpyrrolidone in tailoring polyamide layer for high-performance nanofiltration membranes. *Desalination* **564**, 116767 (2023).
22. Shen, K., Hua, W., Ding, S. & Wang, X. Customizing versatile polyamide nanofiltration membrane by the incorporation of a novel glycolic acid inhibitor. *Sep. Purif. Technol.* **255**, 117632 (2021).
23. Wang, X. *et al.* Poly(caffeic acid) as interlayer to enhance nanofiltration performance of polyamide composite membrane. *Desalination* **545**, 116168 (2023).
24. Shi, Y. *et al.* Nanomorphogenesis of template-induced crumpled polyamide nanofiltration membranes. *J. Membr. Sci.* **686**, 121997 (2023).
25. Liang, Y. *et al.* Polyamide nanofiltration membrane with highly uniform sub-nanometre pores for sub-1 Å precision separation. *Nat. Commun.* **11**, 2015 (2020).
26. Sarkar, P., Modak, S. & Karan, S. Ultraselective and highly permeable polyamide nanofilms for ionic and molecular nanofiltration. *Adv. Funct. Mater.* **31**, 1–8 (2021).
27. Zhou, Z. *et al.* Ultrathin polyamide membranes enabled by spin-coating assisted interfacial polymerization for high-flux nanofiltration. *Sep. Purif. Technol.* **288**, 120648 (2022).
28. Zhu, J. *et al.* Rapid water transport through controllable, ultrathin polyamide nanofilms for high-performance nanofiltration. *J. Mater. Chem. A* **6**, 15701–15709 (2018).
29. Liu, Z. *et al.* Highly anions-selective polyamide nanofiltration membrane fabricated by rod-coating assisted interfacial polymerization. *J. Membr. Sci.* **668**, 121273 (2023).
30. Zhu, C.-Y. *et al.* Vacuum-assisted diamine monomer distribution for synthesizing polyamide composite membranes by interfacial polymerization. *J. Membr. Sci.* **616**, 118557 (2020).

31. Yang, S. *et al.* Electrosprayed polyamide nanofiltration membrane with intercalated structure for controllable structure manipulation and enhanced separation performance. *J. Membr. Sci.* **602**, 117971 (2020).
32. Zheng, Y., Wu, Y., Zhang, B. & Wang, Z. Preparation and characterization of CO_2-selective Pebax/NaY mixed matrix membranes. *J. Appl. Polym. Sci.* **137**, 48398 (2020).
33. Somrani, A., Hamzaoui, A. H. & Pontie, M. Study on lithium separation from salt lake brines by nanofiltration (NF) and low pressure reverse osmosis (LPRO). *Desalination* **317**, 184–192 (2013).
34. Wang, L., Lin, Y., Tang, Y., Ren, D. & Wang, X. Fabrication of oppositely charged thin-film composite polyamide membranes with tunable nanofiltration performance by using a piperazine derivative. *J. Membr. Sci.* **634**, 119405 (2021).
35. Li, X. *et al.* Preparation and characterization of positively charged polyamide composite nanofiltration hollow fiber membrane for lithium and magnesium separation. *Desalination* **369**, 26–36 (2015).
36. Jiang, C. *et al.* Poly(piperazine-amide) nanofiltration membrane with innate positive charge for enhanced bivalent cation rejection and mono/bivalent cation selectivity. *J. Membr. Sci.* **664**, 121060 (2022).
37. Xu, P. *et al.* Positive charged PEI-TMC composite nanofiltration membrane for separation of Li^+ and Mg^{2+} from brine with high Mg^{2+}/Li^+ ratio. *Desalination* **449**, 57–68 (2019).
38. Li, Y. *et al.* Fabrication of positively charged nanofiltration membrane with uniform charge distribution by reversed interfacial polymerization for Mg^{2+}/Li^+ separation. *J. Membr. Sci.* **659**, 120809 (2022).
39. Xu, P. *et al.* Fabrication of highly positively charged nanofiltration membranes by novel interfacial polymerization: Accelerating Mg^{2+} removal and Li^+ enrichment. *J. Membr. Sci.* **668**, 121251 (2023).
40. Zhang, S. *et al.* Bifunctional polyhedral oligomeric silsesquioxane engineered polyamide membrane for efficient Li^+/Mg^{2+} separation. *Sep. Purif. Technol.* **327**, 124875 (2023).
41. Guo, C. *et al.* One-step construction of the positively/negatively charged ultrathin Janus nanofiltration membrane for the separation of Li^+ and Mg^{2+}. *ACS Appl. Mater. Interfaces* **15**, 4814–4825 (2023).
42. Zhang, T. *et al.* Advanced Mg^{2+}/Li^+ separation nanofiltration membranes by introducing hydroxypropyltrimethyl ammonium chloride chitosan as a co-monomer. *Appl. Surf. Sci.* **616**, 156434 (2023).
43. Hu, P. *et al.* Modification of polyamide nanofiltration membrane with ultra-high multivalent cations rejections and mono-/divalent cation selectivity. *Desalination* **527**, 115553 (2022).
44. Shen, Q., Xu, S., Xu, Z., Zhang, H. & Dong, Z. Novel thin-film nanocomposite membrane with water-soluble polyhydroxylated fullerene for the separation of Mg^{2+} /Li^+ aqueous solution. *J. Appl. Polym. Sci.* **136**, (2019).
45. Zhao, J. *et al.* Mix-charged polyamide membranes via molecular hybridization for selective ionic nanofiltration. *J. Membr. Sci.* **644**, 120051 (2022).

46. Zhang, S. *et al.* Guanidyl-incorporated nanofiltration membranes toward superior Li^+/Mg^{2+} selectivity under weakly alkaline environment. *J. Membr. Sci.* **663**, 121063 (2022).
47. Yuan, B. *et al.* Aliphatic polyamide nanofilm with ordered nanostripe, synergistic pore size and charge density for the enhancement of cation sieving. *J. Membr. Sci.* **660**, 120839 (2022).
48. Han, Q., Liu, D., Huang, X., Xie, Q. & Meng, J. Composite membrane of polyguanidine cationic surface for desalination. *Water Supply* **22**, 4798–4809 (2022).
49. Huang, B.-Q. *et al.* Dually charged polyamide nanofiltration membranes fabricated by microwave-assisted grafting for heavy metals removal. *J. Membr. Sci.* **640**, 119834 (2021).
50. Lu, D. *et al.* Constructing a selective blocked-nanolayer on nanofiltration membrane via surface-charge inversion for promoting Li^+ permselectivity over Mg^{2+}. *J. Membr. Sci.* **635**, 119504 (2021).
51. Li, L. *et al.* Polyethyleneimine modified polyamide composite nanofiltration membrane for separation of lithium and magnesium. *J. Water Process Eng.* **54**, 103894 (2023).
52. Wu, H. *et al.* A novel nanofiltration membrane with [MimAP][Tf_2N] ionic liquid for utilization of lithium from brines with high Mg^{2+}/Li^+ ratio. *J. Membr. Sci.* **603**, 117997 (2020).
53. Li, H. *et al.* Improving Mg^{2+}/Li^+ separation performance of polyamide nanofiltration membrane by swelling-embedding-shrinking strategy. *J. Membr. Sci.* **669**, 121321 (2023).
54. Xu, Y. *et al.* High performance Mg^{2+}/Li^+ separation membranes modified by a bis-quaternary ammonium salt. *Desalination* **526**, 115519 (2022).
55. Soyekwo, F., Wen, H., Liao, D. & Liu, C. Fouling-resistant ionic graft-polyamide nanofiltration membrane with improved permeance for lithium separation from $MgCl_2$/LiCl mixtures. *J. Membr. Sci.* **659**, 120773 (2022).
56. Feng, Y., Peng, H. & Zhao, Q. Fabrication of high performance Mg^{2+}/Li^+ nanofiltration membranes by surface grafting of quaternized bipyridine. *Sep. Purif. Technol.* **280**, 119848 (2021).
57. Gu, T. *et al.* Quaternary ammonium engineered polyamide membrane with high positive charge density for efficient Li^+/Mg^{2+} separation. *J. Membr. Sci.* **659**, 120802 (2022).
58. Wang, M. *et al.* Synergistic regulation of macrocyclic polyamine-based polyamide nanofiltration membranes by the interlayer and surfactant for divalent ions rejection and mono-/di-ions sieving. *Desalination* **544**, 116131 (2022).
59. Yuan, B. *et al.* Polyamide nanofiltration membrane fine-tuned via mixed matrix ultrafiltration support to maximize the sieving selectivity of Li^+/Mg^{2+} and Cl^-/SO_4^{2-}. *Desalination* **538**, 115929 (2022).
60. Chen, K. *et al.* An interlayer-based positive charge compensation strategy for the preparation of highly selective Mg^{2+}/Li^+ separation nanofiltration membranes. *J. Membr. Sci.* **684**, 121882 (2023).
61. Wang, G. *et al.* Cationic COF nanosheets engineered positively charged polyamide membranes for efficient divalent cations removal. *J. Membr. Sci.* **684**, 121863 (2023).
